# Proceedings from the 2024 precision public health research symposium: advancing health equity through precision public health

**DOI:** 10.1186/s12919-025-00331-7

**Published:** 2025-07-15

**Authors:** Allison Olawsky, Samantha Keohane, Megan C. Roberts, Amelia Smit, Cason Whitcomb, Dana Lee Olstad, Erin Turbitt, Jarrod Marable, Julia Steinberg, Kate Saylor, Kim Foss, Latrice Landry, Caitlin G. Allen

**Affiliations:** 1 Medical University of South Carolina, Charleston, SC USA; 2 University of North Carolina, Chapel Hill, NC USA; 3 University of Sydney, Camperdown, NSW Australia; 4 University of Calgary, Calgary, AB Canada; 5 University of Technology Sydney, Ultimo, NSW Australia; 6 The Daffodil Centre, The University of Sydney, a joint venture with Cancer Council NSW, Sydney, Australia; 7 University of Pennsylvania, Philadelphia, PA USA; 8 Department of Implementation Science, Wake Forest University School of Medicine, Winston Salem, United States

## Introduction

While precision public health (PPH) has emerged as a population-level approach that allows for tailoring of prevention and health promotion strategies by delivering the right intervention to the right population at the right time, concerns have been raised about whether this type of approach may exacerbate health inequalities. Key challenges include an overemphasis on individual agency to accomplish precision-based prevention activities and potential to divert limited resources away from established drivers of public health. Another challenge is limitations to risk prediction models due to limited data or knowledge, leading to differences in prediction accuracy between subgroups (and thus inequitable benefits from risk prediction), or even potential harms if predictions for some subgroups are inaccurate and misleading.

The Precision Public Health Network (PPHN) has sought to address the critical issues regarding health equity and PPH through several commentaries highlighting opportunities to study the impact of PPH on health equity outcomes and develop frameworks to conceptualize these challenges [1–4]. In 2024, the PPHN brought together researchers to further discuss these topics and hear examples of how PPH can help address health equity issues. As part of an ongoing series of annual meetings, the PPHN hosted the Precision Public Health Research Symposium with the theme of *Advancing Health Equity through Precision Public Health.* Prior to the 2024 conference, the PPHN hosted virtual meetings and workshops including the inaugural 2021 meeting and 2023 conference focused on *Applying Implementation Science to Precision Public Health.* Through these meetings, the PPHN provides opportunities for knowledge sharing, networking, and sustained collaborations focused on key issues in the field of PPH.

This conference proceedings paper describes our approach to organizing the PPHN conference focused on health equity, summarizes speakers presentations, and provides key take-aways. The conference agenda and recorded presentations are available at Precision Health Research Symposium | MUSC Research.

## Methods

The in-person conference was organized by a planning committee with funding from R13CA261703. This was the first in-person conference hosted by the Precision Public Health Network. The international planning committee met virtually on a monthly basis to plan the meeting. The committee developed the program and speaker list, planned the process for poster submissions and selection, and organized a process for identifying lightning talk speakers. Abstracts were solicited for the poster session and lightning talks, reviewed and scored by the planning committee based on relevance to the conference theme, quality of content, and contribution to science. Advertising was conducted in collaboration with the host site (Medical University of South Carolina) and included outreach via network listservs, social media, and direct outreach via social media posts and speaker highlights leading up to the event.

Conference attendees received an evaluation form via QR code and email after the conference. The evaluation included questions about the overall quality of the meeting, as well as ratings of each session.

## Results

On November 15, 2024, we hosted a hybrid conference with 101 participants, including 11 who attended virtually (Table 1). The theme of the event was *Advancing Health Equity through Precision Public Health*. Of the registrants, 95.7% were from North America (*n* = 201), followed by 2.9% from Asia Pacific (*n* = 6), 1.4% from Europe (*n* = 3), and 0.5% from Africa (*n* = 1). Participants represented various public health disciplines, including health services research (33.8%), health behavior (13.8%), health policy (11.0%), epidemiology (10.5%), biostatistics (9.5%), and environmental health (2.9%), while 19.0% did not specify their focus. There were a total of 19 posters.

**Table 1:** Conference Participant Information

	**Attendees (*n* = 101)**
**Geography**	
North America	99 (98.0%)
Asia Pacific	2 (2.0%)
Europe	0 (0.0%)
Africa	0 (0.0%)
**Public Health Discipline**	
Health Services Research	27 (26.7%)
Health Behavior	10 (9.9%)
Health Policy	5 (5.0%)
Epidemiology	5 (5.0%)
Biostatistics	5 (5.0%)
Environmental Health	2 (2.0%)
Unspecified	47 (46.5%)

^*^Public health disciplines were self-reported by participants

The agenda featured six invited speakers with expertise in key areas of precision public health such as genetics, biomedical informatics, nutrition, health services research, public health sciences, and epidemiology. Additionally, five lightning talks showcased work selected from abstracts by students and clinicians from fields including pediatrics, public health sciences, genetics, and biomedical informatics. A community panel highlighted the importance of involving marginalized groups in precision health initiatives, bringing together researchers, clinicians, advocates, and community members to discuss inclusive strategies.

The conference concluded with key takeaways and actionable next steps shared during both the morning and afternoon sessions, reinforcing the commitment to advancing precision public health through collaboration and community engagement.

### Caitlin Allen: Upstream and downstream exclusivity in research inclusion

Dr. Caitlin Allen explored the concept of exclusivity in research, focusing on the barriers that restrict certain groups from participating and benefiting from scientific advancements. She described upstream exclusivity as the limited access marginalized communities face in becoming part of research studies. Equally significant is downstream exclusivity, where obstacles—both material and psychological—impede these same communities from accessing the benefits of scientific discoveries. Allen emphasized the need to dismantle these barriers to foster inclusivity, ensuring that scientific progress is not only equitable but also reflective of diverse populations.

### Paul Heider: Behavioral testing for algorithmic bias

Paul Heider discussed the critical role of behavioral testing in identifying and mitigating algorithmic bias within natural language processing (NLP) models used in healthcare. His work revealed how clinical notes containing pejorative terms often lead to underdiagnosis, particularly for patients from marginalized groups. Heider underscored the importance of re-educating physicians to improve the quality of data input while simultaneously refining algorithms to account for harmful biases. His takeaway was that avoiding bias requires vigilance in both the data fed into models and in the models' design, to ensure equitable healthcare outcomes through careful, ethical AI development.

### Lior Rennert: Statewide network for outbreak detection

Lior Rennert described his work in developing a statewide network for outbreak detection and emergency response, focusing on medically underserved communities in South Carolina. Collaborating with Prisma Health and MUSC, Rennert emphasized the necessity of real-time data from sources like electronic health records (EHRs), digital traces, and wastewater to forecast outbreaks and allocate resources efficiently. He detailed how mobile health units were deployed during the COVID-19 pandemic to reach high-risk areas, stressing the importance of GIS mapping to identify hotspots and optimize interventions. His vision extended beyond infectious diseases, aiming to apply this framework to broader public health initiatives for more targeted and timely responses.

### Guilherme Del Fiol: GARDE Platform for hereditary cancer syndromes

Guilherme Del Fiol presented the GARDE platform, designed to identify individuals at risk for hereditary cancer syndromes, emphasizing the urgency of early detection. He explained how the GARDE algorithm integrates with EHRs to automate the process of identifying patients eligible for genetic testing, streamlining the pathway from risk identification to genetic counseling. Del Fiol shared insights from the BRIDGE trial, where automated chatbot interventions were as effective as standard care genetic counseling but far more scalable. He highlighted the platform's adaptability, which allows it to be used for other health interventions, such as tobacco cessation and chronic pain management, making GARDE a versatile tool in precision health.

### Deborah Tate: Precision nutrition and public health

Deborah Tate’s talk centered on the need for precision nutrition to address the diverse responses individuals have to dietary interventions. She explained how the Nutrition for Precision Health (NPH) study, an extension of the All of Us research initiative, aims to tailor dietary recommendations based on genetic, cultural, and environmental factors. Tate discussed how personalized messaging and adaptive interventions are critical in promoting adherence to healthier diets. She highlighted the potential of passive data collection, such as glucose monitoring, to provide real-time feedback, underscoring the need for individualized approaches to public health messaging and nutrition guidance to promote sustained health improvements.

### Dayanna Ramirez Leon: Addressing disparities in genetic testing

Dayanna Ramirez Leon explored the significant disparities in genetic testing, particularly within the Latino community. According to the Health Information National Trends Survey 6 (2022), **51% of individuals eligible for genetic testing were unaware of their risk**, and another **40% were aware but not offered testing**. She emphasized that traditional health behavior models, such as the Health Belief Model, failed to predict genetic testing behaviors in Latinos. Ramirez Leon argued that culturally relevant adjustments to these models are necessary, considering the unique cultural values, family dynamics, and evolving identity within Latino communities. Her conclusion underscored the importance of culturally tailored approaches to increase awareness and access to genetic testing.

### Grace Leon-Lozano: Underrepresentation in clinical research

Grace Leon-Lozano focused on the underrepresentation of Latinos in clinical research, highlighting the need for tailored dissemination strategies. She emphasized the importance of diversity within groups traditionally considered homogeneous and advocated for age-based genomic screening programs tailored to specific communities. Leon-Lozano pointed out that linguistic diversity is critical, noting that current automated translation tools are insufficient. She stressed the need for diverse advisory boards to ensure messaging is both accurate and meaningful, suggesting that human translators, reviewed by community members, are essential to bridge the communication gap effectively.

### Kristen Lancaster: Rapid genomic sequencing in pediatric patients

Kristen Lancaster presented on the transformative potential of ultra-rapid genomic sequencing for hospitalized pediatric patients, where results can be available within 2–5 days. This rapid sequencing aids in diagnosing rare and actionable conditions, leading to significant changes in medical management for 19 patients, with 26% experiencing changes in diet or medication and 21% in palliative care adjustments. Lancaster noted that exome sequencing is especially relevant for cases involving developmental delays or congenital anomalies. She emphasized that early, accurate diagnosis through rapid sequencing can significantly impact clinical outcomes and improve patient care.

### Alison Fohner: Phenomenological approaches to equity in electronic health research

Alison Fohner posed a critical question: Does Community-Based Participatory Research (CBPR) improve the health of communities not directly participating in the research? She advocated for a phenomenological approach, emphasizing the importance of ensuring that outlier data points—often dismissed as errors—are not excluded prematurely. Fohner called for a more careful assessment of such data to avoid inadvertently excluding marginalized populations from research benefits. Her conclusion was a call to ensure research efforts improve health equity both within and beyond participating communities.

### Pamela Ganschow: Cancer prevention and early detection in underserved communities

Pamela Ganschow addressed the widespread lack of awareness regarding hereditary cancer risks, noting that more than 80% of individuals with genetic mutations remain unaware of their risk. Since 2005, the USPSTF has recommended family history-based screening for hereditary cancer syndromes, yet significant racial and socioeconomic disparities persist. Ganschow identified several barriers, including limited access to testing, provider bias, and low referral rates, and proposed solutions such as implementing universal screening approaches in Federally Qualified Health Centers (FQHCs), improving provider training, and developing scalable, sustainable alternative care models.

### Jada Hamilton: Mainstreaming hereditary cancer genetic testing for health equity

Jada Hamilton highlighted the inequities in hereditary cancer genetic testing through a mainstreaming model designed to enhance scalability and accessibility. Her research demonstrated that 10% of participants had pathogenic variants (PV) and 16% had variants of uncertain significance (VUS). Despite high satisfaction rates with pre-test education, knowledge retention remained low until supplemented by genetic counseling. Hamilton emphasized the factors leading to disparities in testing access, including language barriers, mistrust, and limited dissemination of genomic information among families. She described the NCI-funded Imagine project, which developed linguistically and culturally appropriate educational materials for Spanish and Creole-speaking communities, ensuring accessibility at a 7th-grade reading level. Hamilton advocated for integrating genetic testing more seamlessly into primary care to reduce disparities and improve health outcomes.

## Evaluation

Forty-six individuals completed the online evaluation after the conference (Table 2). Respondents had high rankings of the overall meeting experience (on a 5-point Likert scale, with 1 being lowest and 5 being highest/best rating). The overall quality of the event was rated 4.58, with high likelihood of recommending future research events to others (4.69) and high likelihood of attending the next sponsored event (4.46). The overall quality of facilities (4.64), overall quality of speakers (4.61), overall quality of poster sessions (4.38), and overall meeting experience (4.55) were all highly ranked. The relatively lowest scored component of the meeting experience was the availability of networking (4.10). Individuals also strongly agreed that the conference met its standards (4.67), they were satisfied with the overall conference experience (4.72), and they were more likely to engage in precision public health after attending (4.69).

**Table 2:** Overall meeting experience (*n* = 46)

	**Mean**	**Standard Deviation**
Overall Quality of Event	4.58	0.59
Likelihood of Recommending MUSC Research Events to Others	4.69	0.52
Likelihood of Attending the Next MUSC Research Sponsored Symposium	4.46	0.78
Overall Quality of Facilities	4.64	0.85
Overall Quality of Speakers	4.61	0.83
Overall Quality of Poster Session	4.38	0.84
Overall Availability of Networking	4.1	1.02
Overall Meeting Experience	4.55	0.64
I Feel That the Conference Met Its Standards^a^	4.67	0.62
Satisfaction of Overall Conference Experience^a^	4.72	0.6
I Am More Likely to Engage in Precision Public Health after Attending this Symposium^a^	4.69	0.47

Likert Scale: 1 = Poor, 2 = Fair, 3 = Good, 4 = Very Good, 5 = Excellent

^a^Likert Scale: 1 = Strongly Disagree, 2 = Disagree, 3 = Neither agree or disagree, 4 = Agree, 5 = Strongly Agree

The evaluation also assessed the quality of speakers. Twenty-six individuals responded to these questions. We asked about each speaker individually and data were aggregated across sessions. Overall, we received high ratings for the overall quality of information provided (4.58), quality of visual aids (4.51), presenter’s knowledge (4.66), presenter’s presentation skills (4.56), and quality of overall presentation (4.56). There were high levels of agreement with statements of: this session advanced my understanding (4.62) and this session covered relevant materials (4.48) **(**Table 3).

**Table 3:** Opening and Speaker Sessions (*n* = 26)

	**Mean**	**Standard Deviation**
Quality of Information Provided	4.58	0.64
Quality of Visual Aids	4.51	0.71
Presenter's Knowledge of the Subject	4.66	0.59
Presenter's Presentation Skills	4.56	0.65
Quality of Overall Presentation	4.56	0.64
This Session Advanced My Understanding^a^	4.62	0.53
This Session Covered Relevant Material^a^	4.48	0.8

Likert Scale: 1 = Poor, 2 = Fair, 3 = Good, 4 = Very Good, 5 = Excellent

^a﻿^Likert Scale: 1 = Strongly Disagree, 2 = Disagree, 3 = Neither agree or disagree, 4 = Agree, 5 = Strongly Agree

## Discussion

The 2024 Precision Public Health Research Symposium: *Advancing Health Equity through Precision Public Health* convened 101 individuals in person to discuss issues of health equity and PPH. Key takeaways from the meeting centered on advancing health equity through inclusive research practices, ethical technology development, and culturally responsive interventions. A major theme was the need to address both upstream and downstream barriers to participation in research and healthcare, ensuring that marginalized communities have access to both the research opportunities and scientific advancements. Specific examples included addressing disparities in genetic testing and hereditary cancer screening by addressing systematic barriers, provider bias, access issues, and culturally tailored education strategies. Additionally, several speakers addressed the role of AI and algorithm bias, highlighting the need for vigilance in data collection, training, and ethical AI development to prevent biased clinical decision-making. Opportunities to advance these initiatives include public health surveillance and emergency response efforts that demonstrate the importance of real-time data integration and targeted outreach. Overall, the discussions reinforced the need for systemic changes that prioritize health outcomes and reinforced the need for systemic changes to prioritize equity, community engagement, and scalable innovations to reduce health disparities. Future conferences and network activities will build from this meeting and focus on other key topics, including capacity building for PPH, ethical legal and social implications of precision public health, leveraging AI and digital tools for PPH, community-driven approaches for PPH, policy and practice implications of PPH, and advanced implementation science in PPH.

## Data Availability

Data are available upon request to the corresponding author.
